# Prognostic role of extracellular matrix metalloproteinase inducer/CD147 in gastrointestinal cancer: a meta-analysis of related studies

**DOI:** 10.18632/oncotarget.12745

**Published:** 2016-10-19

**Authors:** Xiaohui Huang, Weisong Shen, Hongqing Xi, Kecheng Zhang, Jianxin Cui, Bo Wei, Lin Chen

**Affiliations:** ^1^ Department of General Surgery, Chinese People's Liberation Army General Hospital, Beijing 100853, China; ^2^ Department of General Surgery, Jinling Hospital, Medical School of Nanjing University, Nanjing 210002, China

**Keywords:** gastrointestinal cancer, CD147, prognosis, meta-analysis

## Abstract

The prognostic role of Extracellular matrix metalloproteinase inducer (EMMPRIN/ CD147) in gastrointestinal cancer remains controversial. We systematically reviewed the evidence of assessment of CD147 expression in gastrointestinal cancer to help clarify this issue. Pubmed, Embase, Cochrane Library and Web of Science databases were searched to identify eligible studies to evaluate the association of CD147 expression and disease-free and overall survival of gastrointestinal cancer. Hazard ratios (HRs) were pooled to estimate the effect. CD147 overexpression was significantly correlated with poor disease-free survival (HR 2.38, 95% CI 1.43–3.97) and overall survival (HR 1.64, 95% CI 1.25–2.14) of cancer patients. Furthermore, CD147 overexpression was significantly association with TNM stage (TIII/TIV vs TI/TII: OR 3.60, 95% CI 1.85–7.01), the depth of invasion (T3/T4 vs T1/T2: OR 2.04, 95% CI 1.25–3.33), lymph node metastasis (positive vs negative: 2.35, 95% CI 1.14–4.86), distant metastasis (positive vs negative: OR 4.78, 95% CI 1.43–16.00). Our analyses demonstrate that CD147 was effectively predictive of worse prognosis in gastrointestinal cancer. Moreover, Identifying CD147 may help identify new drug targets for cancer therapy.

## INTRODUCTION

Gastrointestinal cancers are the main malignant tumors in both men and women worldwide [1, 2]. Although progression has been made in its diagnosis and treatment, some gastrointestinal cancers are still incurable. This is largely because most patients are at an advanced stage at the time of diagnosis, so have a correspondingly poor prognosis. Disease prognosis correlates directly with the incidence of carcinoma relapse, which is mainly caused by early metastasis. Therefore, the prediction of tumor relapse and the prevention of early metastasis is a promising therapy for gastrointestinal cancer. It is therefore very important to confirm prognostic markers for gastrointestinal cancer which can help identify better preventive methods for gastrointestinal cancer patients.

Several molecular markers, such as matrix metalloproteinase (MMPs), vascular endothelial growth factor (VEGF), E-cadherin, and epidermal growth factor, have been proven to associate with prognosis for carcinoma patients [3–6]. Among the biomarkers, the expression of extracellular matrix metalloproteinase inducer (CD147) has attracted interest. CD147 is one of cell glycoprotein of immunoglobulin super family. It is highly expressed in malignant carcinomas and is a prognosis marker of cancer progression [7]. CD147 arouses normal cells to express MMPs, which are a group of zinc-dependent proteins that degrade the expression of extracellular matrix (ECM) [8, 9]. Low expression of the ECM surrounding primary tumors is essential for carcinoma invasion and metastasis [8].

As well as the important function of CD147 in tumor progression, its role in carcinoma invasiveness was also identified in several malignancies. Some reports demonstrated that CD147 expression was correlated with the prognosis of various human carcinomas [10–15]. CD147 has also been reported to be associated with tumor progression, metastasis, relapse, and prognosis of gastrointestinal carcinoma [16–22]. However, not all findings are consistent. Meta-analysis is a powerful method that overcomes the deficiency of small sample sizes of a individual centre [23]. Thus, we perform the meta-analysis to evaluate the prognostic significance of CD147 in gastrointestinal cancer.

## RESULTS

### Study selection and characteristics

**Figure 1:**
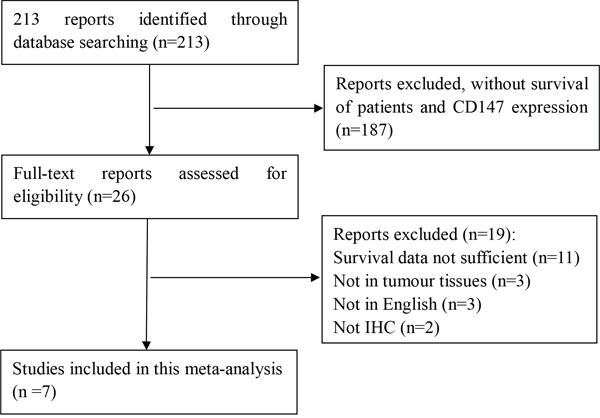
Flow chart of studies included

A total of 213 researches were retrieved by the search strategies described. After browsing the abstract and full-text, seven studies were into meta-analysis. All studies met the inclusion criteria and quality assessment standard. The selection process is shown in Figure 1, and the main characteristics of the seven studies are summarized in Table 1. Three studies were of gastric cancer, and four were of colorectal cancer. Three studies evaluated patients from China one from Korea, one from Norway, one from Germany,, and one from Japan. The seven studies consisted of a total of 1,993 patients, with sample sizes varying from 210 to 436 patients.

**Table 1:** Major features of the included studies

Study	Year	Study location	Number of patients	Races	Malignant disease	Technology	Detected sample	Antibody	Staining patterns	Cut-off	CD147 expression(%)
**Zheng [16]**	2006	Japan	234	Asian	Gastric cancer	TMA and IHC	Tissue	Mouse anti-EMMPRIN	Membrane and cytoplasm	P>5%	64.96%
**Zhang [17]**	2012	China	436	Asian	Gastric cancer	TMA and IHC	Tissue	Mouse monoclonal antibody	Membrane	S≥4	66.51%
**Chu [18]**	2014	China	223	Asian	Gastric cancer	IHC	Tissue	Mouse anti-human CD147 monoclonal antibody	Membrane and cytoplasm	S≥1	59.64%
**Boye [19]**	2012	Norway	277	Caucasian	Colorectal cancer	IHC	Tissue	Goat polyclonal anti-EMMPRIN antibody	Membrane and cytoplasm	P≥5%	71.48%
**Zhu [20]**	2013	China	328	Asian	Colorectal cancer	TMA and IHC	Tissue	Mouse anti-human HAb18G/CD147 monoclonal antibody	Membrane and cytoplasm	S≥1	63.11%
**Stenzinger [21]**	2011	Germany	285	Caucasian	Colorectal cancer	TMA and IHC	Tissue	Rabbit polyclonal antibody	Membrane and cytoplasm	S>1	49.82%
**Jung [22]**	2011	Korea	210	Asian	Colorectal cancer	TMA and IHC	Tissue	Monoclonal antibody	Membrane and cytoplasm	S≥3	62%

TMA, tissue microarray; IHC, immunohistochemistry; P, proportion of stained tumor cells; S, scores for intensity and proportion of stained tumor cells.

### The association between CD147 and survival in gastrointestinal cancer

**Figure 2:**
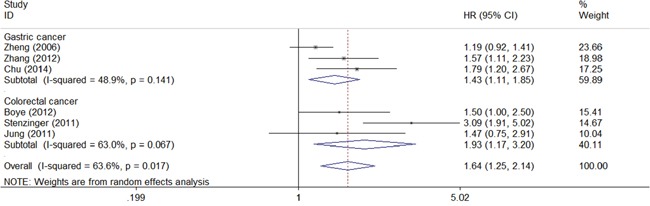
Meta-analysis of impact of CD147 expression on overall survival of patients Results are presented as individual and pooled HR, and 95% CI.

CD147 in gastrointestinal cancer tissues was shown to be significantly associated with poor overall survival of cancer patients (combined HR 1.64, 95% CI 1.25–2.14). Subgroup analysis by malignant disease also revealed a significant relationship between CD147 and overall survival in gastric cancer (HR 1.43, 95% CI 1.11–1.85) and colorectal cancer (HR 1.93, 95% CI 1.17–3.20) (Figure 2).

**Figure 3:**
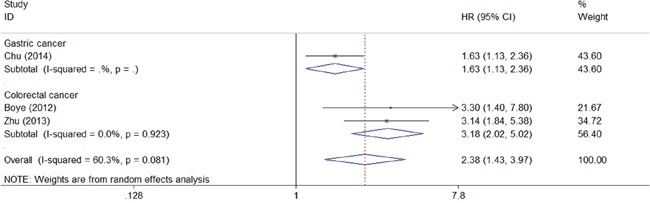
Meta-analysis of impact of CD147 expression on disease-free survival of patients Results are presented as individual and pooled HR, and 95% CI.

We also assessed the association between CD147 and disease-free survival in gastrointestinal cancer. As shown in Figure 3, CD147 expression in gastrointestinal cancer tissues was associated with poor disease-free survival of gastrointestinal cancer (combined HR 2.38, 95% CI 1.43–3.97). Moreover, subgroup analysis showed a significant relationship between CA147 and disease-free survival in both gastric carcinoma (HR 1.63, 95% CI 1.13–2.36) and colorectal carcinoma (HR 3.18, 95% CI 2.02–5.02).

### The association between CD147 and clinical features

**Figure 4:**
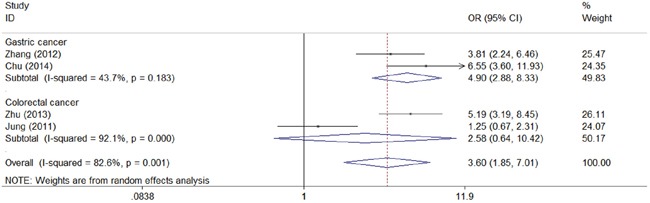
Meta-analysis of impact of CD147 expression on TNM stage of patients Results are presented as individual and pooled OR, and 95% CI.

**Figure 5:**
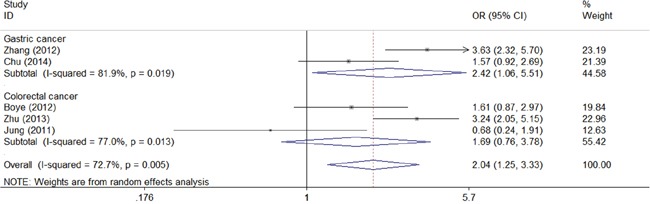
Meta-analysis of impact of CD147 expression on the depth of invasion of patients Results are presented as individual and pooled OR, and 95% CI.

**Figure 6:**
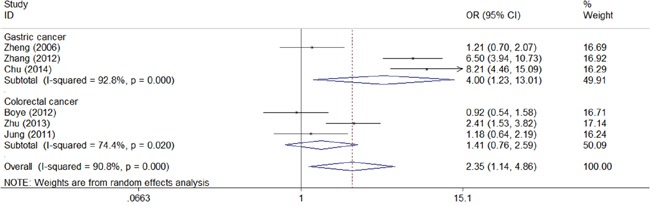
Meta-analysis of impact of CD147 expression on lymph node metastasis of patients Results are presented as individual and pooled OR, and 95% CI.

**Figure 7:**
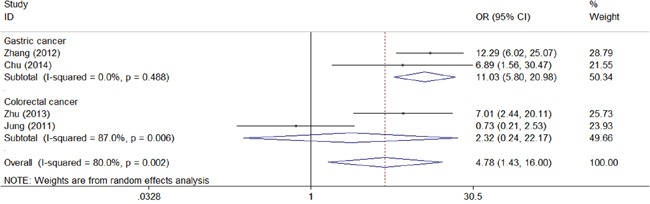
Meta-analysis of impact of CD147 expression on distant metastasis of patients Results are presented as individual and pooled OR, and 95% CI.

We next assessed the association between CD147 and clinical characteristic s of gastrointestinal carcinoma. As shown in the forest plots, CD147 was significantly associated with TNM stage (TIII/TIV vs TI/TII: combined OR 3.60, 95% CI 1.85–7.01) (Figure 4), depth of invasion (T3/T4 vs T1/T2: combined OR 2.04, 95% CI 1.25–3.33) (Figure 5), lymph node metastasis (positive vs negative: combined OR 2.35, 95% CI 1.14–4.86) (Figure 6), and distant metastasis (positive vs negative: combined OR 4.78, 95% CI 1.43–16.00) (Figure 7).

### Publication bias

**Figure 8:**
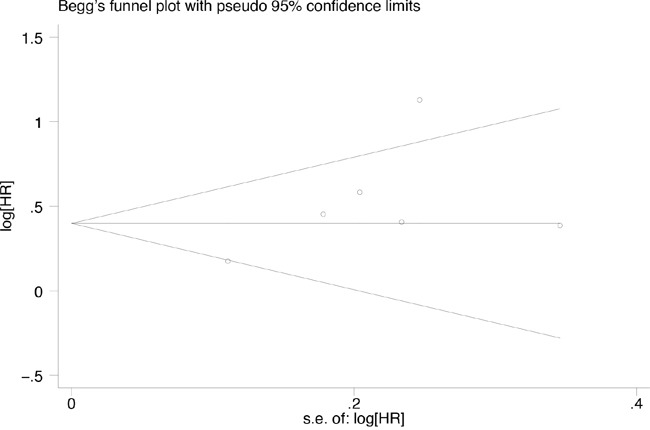
Funnel plot for the evaluation of potential publication bias in the impact of CD147 expression on overall survival

Publication bias was assessed based on overall survival using Begg's test. No publication bias was identified in these studies (p = 0.573). Similarly, the funnel plots for publication bias revealed a degree of symmetry (Figure 8).

## DISCUSSION

CD147 is abundantly expressed in various types of cancer tissue, and may promote cancer metastasis by regulating the processes of cellular substrate and adhesion [24–26]. CD147 also plays a key role in degradation of the ECM through MMP induction [27–29], regulates VEGF expression [30], stimulates proliferation, migration, and cell survival independently of MMPs [31], and leads to multidrug resistance to some chemotherapeutic drugs [32].

To our knowledge, this is the first meta-analysis to assess the association between CD147 expression and disease-free and overall survival of gastrointestinal cancer patients. Our meta-analysis combined the results of 1,993 gastrointestinal cancer patients from seven studies. The meta-analysis indicated that CD147 expression can be used to significantly predict poor disease-free and overall survival of gastrointestinal cancer patients. Subgroup analysis showed that the high expression of CD147 can predict poor prognosis in both gastric cancer and colorectal cancer.

The tissue invasion, lymphatic metastases and distant metastases of carcinomas, are the major prognostic factors of solid cancers [21]. Traditional clinical characteristics, such as size and stage of gastrointestinal cancer, can predict clinical survival of patients. In our study, significant correlations were observed between CD147 and clinical characteristics including the depth of invasion, lymph node metastasis, TNM stage, and distant metastasis.

Our meta-analysis has a number of advantages. First, it rigorously adhered to reporting recommendations for tumor marker prognostic studies (REMARK) guidelines, and carefully searched relevant studies from PubMed, Embase, Cochrane Library, and Web of Science databases. Second, the enrolled researches all had a satisfactory quality based on the strict inclusion criteria. Third, subgroup analysis effectively minimized heterogeneity of the included studies and further explored the application of CD147 as a predictive marker for gastric and colorectal cancer. Fourth, Begg's funnel plots indicated that no publication bias existed in the meta-analysis. This reflected our methodological assessment of the researches to avoid selection biases. Our comprehensive literature search method also minimized publication bias.

Nevertheless, our meta-analysis has some limitations. First, it included just seven studies, resulting in a relative insufficiency in the subgroup analysis, particularly when there was only one study to represent results. Second, the method of calculating HRs from reports might have led to heterogeneity. The HR of one enrolled study was calculated by the method previously documented by Tierney et al [22]. This reported the number of death events of patients and the log-rank data or p-value, which enable the HR to be approximated. Although the calculated HR might be not as reliable as one retrieved from reported studies, we did not find any major deviation of results when comparing our calculated HR with the statistical significance of the study itself. Third, only a small number of included patients were enrolled in our meta-analysis compared with other meta-analyses. Therefore, further study about CD147 is essential to indentify the prognostic role of CD147.

Because of the significant heterogeneity of the enrolled studies, we adopted random effects models when pooling data. Subgroup analysis revealed that the heterogeneity may be due to the difference in malignant disease. The features of cancers might differ based on diverse tumor locations. We conducted subgroup analysis according to malignant disease, and the pooled analysis of gastric cancer and colorectal cancer indicated that the high expression of CD147was associated with poor survival.

Identifying a role for CD147 in driving tumor invasion and metastasis not only helps us understand this biomarker of cancer progression, but also develop new drug target. Our meta-analysis indicates that CD147 expression predicts a bad survival in gastrointestinal carcinomas.CD147 can therefore be used as a prognostic marker for gastrointestinal cancer, and as a predictor of cancer relapse.

## MATERIALS AND METHODS

### Literature search and search strategy

We conducted this meta-analysis following the PRISMA statement [33]. A literature search was performed in Pubmed, Embase, Cochrane Library and Web of Science databases for clinical research published before June 2016 that that assessed CD147 as a prognostic factor for survival of patients with gastrointestinal cancer using immunohistochemistry. The search used the following terms: “gastrointestinal cancer”, “CD147” OR “EMMPRIN” and “prognosis” OR “prognostic” OR “survival”. The references of all relevant articles were evaluated to find other related studies. Article language was limited to English. Two reviewers independently assessed the eligibility of the studies. Agreement was reached for the discrepancies by discussion.

### Inclusion and exclusion criteria

Inclusion criteria: (1) Study evaluated the correlation between CD147 expression and survival of gastrointestinal cancer patients; (2) Study assessed CD147 expression in tumor tissues using immunohistochemistry; (3) If the same team published more than one article, only the most recent or detailed study were included; (4) Study provided sufficient information allowing for estimation of hazard ratios (HRs) and their 95% confidence intervals (CIs); (5) Study was published as a full text in the English language. Exclusion criteria: Impossible to extract effective data from the study's defined clinical outcomes.

### Quality assessment of included studies

Quality assessment and methodological assessment was peer-reviewed by 2 reviewers (Xiaohui Huang and Weisong Shen) independently in each of the acceptable studies, who scored them by the ELCWP scale and REMARK guidelines [34]. This validated quality assessment system is based on four major classifications: scientific design, description of immunohistochemistry methods, generalizability of results, and the analysis of the study data. Each category had a maximal score of 10 points with an overall maximum theoretical score of 40 points. The final scores were presented as percentages. Studies with ≥ 60% points were considered high quality and were included in the meta-analysis. The two investigators reported the quality of included studies independently, and reach a consensus value for each item.

### Data extraction

Two reviewers (Xiaohui Huang and Weisong Shen) independently extracted relevant data. The following data were extracted from each study: first author, year of publication, study period, number of participants, tumor characteristics, survival. HRs and 95% CIs were used to calculate the survival. If HRs and 95% CIs were not directly reported in the included studies, we evaluated the values in the original studies by using the methods illustrated by Tierney et al [35]. If disagreements arose, agreements were reached for the discrepancies by discussion with another author (Bo Wei).

### Statistical analysis

Stata 12.0 software (StatCorp, College Station, TX, USA) was used for statistical analysis. Forest plots were used to estimate the effect of expression on disease-free and overall survival. HR values >1 were judged to indicate a association between CD147 expression and poor outcome. TNM stage (TIII/TIV vs TI/TII), the depth of invasion (T3/T4 vs T1/T2), lymph node metastasis (positive vs negative), and distant metastasis (positive vs negative) were compared using odds ratios (ORs). A p value of <0.05 was regarded as significant. Heterogeneity was assessed by use of Cochran Q and I2 statistics and considered significant at p<0.1. If heterogeneity existed, the analysis used the random effects model. Publication bias was assessed by use of Begg's funnel plot.
